# Effect of circadian rhythm on NAD and other metabolites in human brain

**DOI:** 10.3389/fphys.2023.1285776

**Published:** 2023-11-09

**Authors:** Bernard Cuenoud, Zhiwei Huang, Mickael Hartweg, Mark Widmaier, SongI. Lim, Daniel Wenz, Lijing Xin

**Affiliations:** ^1^ Research and Clinical Development, Nestlé Health Science, Epalinges, Switzerland; ^2^ Department of Medicine, Faculty of Medicine and Health Sciences, Université de Sherbrooke, Sherbrooke, Canada; ^3^ CIBM Center for Biomedical Imaging, Lausanne, Switzerland; ^4^ Animal Imaging and Technology, Ecole Polytechnique Fédérale de Lausanne, Lausanne, Switzerland; ^5^ Clinical Research Unit, Nestlé Research and Development, Lausanne, Switzerland

**Keywords:** NAD, circadian rhythm, brain, MRS, metabolism, lactate, taurine, redox

## Abstract

Nicotinamide Adenine Dinucleotide (NAD) plays a central role in the master circadian clock of the brain (the suprachiasmatic nuclei, SCN) as demonstrated in many model organisms. NAD acts as an enzyme co-factor and substrate and its modulation was found to be tightly regulated to the periodicity of the cycles. However, in human brain, the effect of the circadian rhythm (CR) on the metabolism of the SCN and other brain regions is poorly understood. We conducted a magnetic resonance spectroscopy (MRS) study at a high magnetic field, measuring the occipital brain NAD levels and other metabolites in two different morning and afternoon diurnal states in 25 healthy participants. Salivary cortisol levels were determined to confirm that the experiment was done in two chronologically different physiological conditions, and a behavioral test of risk-taking propensity was administered. Overall, we found that the CR did not significantly affect NAD levels in the occipital brain region. The other brain metabolites measured, including lactate, were not significantly affected by the CR either, except for taurine. The CR did impact risk-taking behavior and salivary cortisol level, confirming that the participants were in two circadian different behavioral and physiological states in the morning and in the afternoon. Measurement of the CR effect on NAD and taurine levels in other brain regions might provide stronger effects.

## 1 Introduction

The circadian rhythm (CR) plays a vital role in regulating cellular, physiological and behavioral processes in health and disease (Greco and Sassone–Corsi, 2019; Reinke and Asher, 2019; Patke et al., 2020; Allada and Bass, 2021). Pre-clinical experiments have shown a direct link between the circadian rhythm and Nicotinamide Adenine Dinucleotide (NAD) levels (Duvilanski et al., 2003), with diminished NAD oscillation amplitude in aging and neurological disease models (Levine et al., 2020). Circadian oscillation of NAD was also observed in human red blood cells, but not in whole blood (O’Neill and Reddy, 2011; Ch et al., 2021). While model organism studies highlight the central role of NAD in the master circadian clock of the brain (the suprachiasmatic nuclei, SCN), no studies have examined the effect of the circadian rhythm on NAD levels in the human brain.

NAD is a crucial cofactor in brain bioenergetics, responsible for metabolism and ATP production, the brain’s energy currency (Lautrup et al., 2019). NAD exists in oxidized (NAD^+^) or reduced (NADH) forms, with the NAD^+^/NADH ratio being important for metabolic balance in the cytosol and mitochondria. NAD^+^ plays a vital role in glycolysis and the TCA cycle by accepting hydride equivalents, forming NADH during ATP production. NADH serves as a central electron donor in mitochondrial oxidative phosphorylation, supplying electrons to the electron transport chain (ETC) for ATP generation. These reactions sustain the high energy demands of the brain primarily through glucose metabolism (Dienel, 2019).

Furthermore, NAD^+^ is a key substrate for various NAD^+^-dependent enzymes involved in genomic stability, mitochondrial homeostasis, stress responses, and cell survival, including Sirtuins (Katsyuba et al., 2020). Modulation of subcellular NAD^+^ synthesis can regulate the timing of signaling pathways. Mammalian circadian rhythms are coordinated with metabolic activity through controlled expression of Nicotinamide phosphoribosyltransferase (NAMPT) (Cambronne and Kraus, 2020). In both the SCN and other circadian tissues, such as liver, NAMPT is directly upregulated by the master regulator CLOCK–BMAL1, a histone acetyl transferase and transcription factor complex (Nakahata et al., 2009; Ramsey et al., 2009). Regulation of NAMPT, in turn, results in oscillating NAD^+^ levels. The rhythmic oscillation of NAD^+^ serves as a feedback ‘timer’ by modulating the activities of NAD^+^-dependent enzymes, including sirtuins (Ramsey et al., 2009; Nakahata et al., 2009; Rutter et al., 2001; Imai and Guarente, 2016), helping to establish the periodicity of the cycles. How the metabolism of the SCN and other brain regions are affected by the CR in human is poorly understood.

Recently, with the development of ^31^P-MRS at high magnetic fields, NAD^+^ and NADH in human brain can be measured non-invasively (Zhu et al., 2015), enabling the monitoring of NAD level and the redox ratio changes. This technique provides a unique non-invasive tool to monitor NAD level and NAD^+^/NADH redox ratio changes in humans under healthy and pathological conditions (Kim et al., 2017; Xin et al., 2018; Cuenoud et al., 2020).

Lactate, an end product of glycolysis in energy metabolism, is produced from pyruvate in a reversible manner by the lactate dehydrogenase enzyme, which requires NAD as a co-factor, and one NADH is converted to NAD^+^ when one pyruvate molecule is converted to lactate. Therefore, the oscillation of NAD may lead to a concomitant fluctuation in lactate from morning to afternoon. Indeed, a 24 h rhythm of lactate was observed in the somatosensory cortex of animals with high lactate levels during the active phase (Shram et al., 2002). The effect of CR on brain metabolites that can be measured by ^1^H-MRS or ^31^P-MRS has been largely unexplored in human.

Under the influence of the CR regulation, behavioral and physiological functions may fluctuate with time during the day (Byrne et al., 2019). This effect has been observed in many cognitive domains, as well as in risky decision making and reward function (Thayer et al., 1988; Logan et al., 2014; Schnell et al., 2014). In a recent review of the clinical literature, it was found that the circadian effect was observed in measures of both reward anticipation and reward receipt, with more consistent evidence for the latter (Byrne et al., 2019). Using the Balloon Analogue Risk Task (BART), a reliable and ecologically valid model for the assessment of individual risk-taking propensity, it has been shown that people typically exhibit a higher reward and risk propensity in the afternoon than in the morning (Byrne and Murray, 2017), which was confirmed in a recent study (Li et al., 2020). Therefore, the BART test offers the opportunity to investigate a possible link between NAD metabolism and behavioral functions in the context of CR regulation, which has not been studied to date.

To explore whether CR could influence the brain NAD level in human, we conducted an MRS study at 7 T, where occipital NAD content, lactate, and other metabolites were assessed in two different morning and afternoon diurnal states in healthy participants. Moreover, the BART test was implemented to explore possible associations between brain NAD and risk-taking behavior. Salivary cortisol levels were determined to confirm that the experiment was done in two circadian different physiological conditions.

## 2 Materials and methods

### 2.1 Participants

Twenty-five healthy participants were recruited for the study and gave informed consent before participating. The study protocol was approved by the local ethics committee in Vaud and registered on ClinicalTrials.gov https://clinicaltrials.gov/ct2/show/NCT05093088.

Inclusion criteria were healthy males (to minimize the influence of potential hormonal heterogeneity on the CR) with age between 18 and 40 years, body Mass Index (BMI) = between 18.5 and 25 kg/m^2^, able to understand and to sign a written informed consent prior to the study, normal or corrected-to-normal vision, completed the Morningness-Eveningness Questionnaire (MEQ) and obtained a score between 30 and 70 (to exclude extreme morning and evening type individuals), no consumption of any beverages or foods with caffeine such as coffee and tea within 24 h prior to and during the experiment, no strenuous exercise the day before and on the day of the study day, completed the 1-week sleeping diary over the week before the experiment showing habitually good sleep, such as falling asleep no later than midnight, waking up no later than 8:00 a.m., and regularly having 7–9 h of sleep every night.

Exclusion criteria were having any metallic, electronic, magnetic, or mechanical implants, devices, or objects, for safety reasons linked to magnetic field exposure, claustrophobia, inability to perform tasks, significant psychological disorders, performing shift work or trans-meridian travel within 1 month before the study initiation, use of medication or nutritional supplements known to affect the circadian system or the NAD levels, hearing disorders, subject having a hierarchical link with the investigator or co-investigators.

### 2.2 Study day experimental design

**FIGURE 1:**
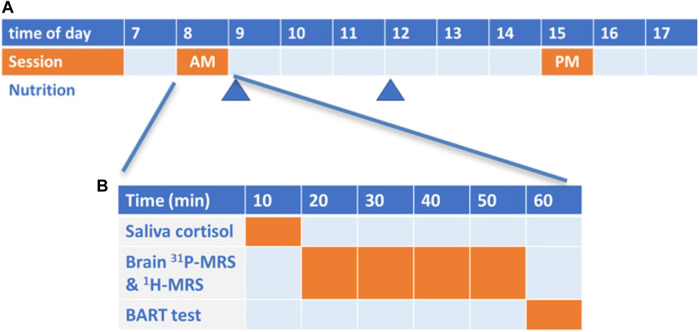
**(A)** Scheme of the overall study (time of the day in hours); **(B)** Scheme of each morning (AM) and afternoon (PM) session.

The study day was carried out in two sessions (Figure 1A). Each participant came to the center in the fasted state (between 8 and 9 a.m.) and the AM session was conducted (Figure 1B). Participant then consumed a standardized breakfast (Boost Plus complete nutrition drink, 360 kcal, Nestlé Health Science) and were provided with a standardized lunch (2 complete nutritional drinks, 720 kcal) to be taken at 12 a.m.-noon. They resumed their daily normal activities and came back to the center in the afternoon (between 3–4 p.m.) for the PM session. The participants were not allowed to consume other food or beverage except for water before the PM session.

Each session lasted about 60 min (Figure 1B) and included a salivary cortisol test (10 min) as an objective biological marker of the circadian rhythms, a brain ^31^P and ^1^H-MRS scan (40 min) to measure various brain metabolites including NAD^+^, NADH and lactate, and a behavioral computerized test of risk taking (10 min) to confirm the effect of CR on participant behavior.

### 2.3 Salivary cortisol

At the beginning of each session, the participant undertook a saliva sampling for cortisol level measurement. The saliva sample was collected (Cortisol-Salivette, Sarstedt), centrifuged at 1,000 rpm for 4 min, and then stored at −20 °C. The samples were shipped on dry ice at the end of the study to laboratory Viollier (Viollier AG, Switzerland, assay #25617) for concentration analysis by Liquid chromatography/mass spectrometry.

### 2.4 ^31^P and ^1^H-magnetic resonance spectroscopy

Brain metabolite measurements were performed on a 7 T/68 cm MR scanner (Siemens Healthineers, Erlangen, Germany) with an in-house-built single-loop ^31^P coil (7 cm diameter) and a ^1^H quadrature surface coil (10 cm diameter) for the coverage of the occipital lobe. B_0_ field inhomogeneity was optimized using first- and second-order shimming with FAST(EST)MAP (Gruetter, 1993). ^31^P-MRS was performed by a pulse-acquire sequence (TR = 3 s, 320 averages, a 200 μs hard pulse, the spectral bandwidth of 6 kHz, 2048 data points) to measure ^31^P metabolites including NADH and NAD^+^. The flip angle was calibrated to maximize the signal of PCr prior to the acquisition protocols. ^1^H-MRS was acquired by a ^1^H quadrature surface coil (10 cm diameter) and a short-TE STEAM sequence (TE/TM/TR = 4.5/25/5,500 ms, 64 averages, voxel size = 35 × 20 × 25 mm^3^) for neurochemical profiling. VAPOR water suppression and outer volume suppression were applied prior to the STEAM localization sequence. T_1_ weighted MR images were obtained by MP2RAGE (TE/TR = 2.31/5,500 ms, TI1/TI2 = 750/2,350 ms, α1/α2 = 4°/5°, FOV = 210 × 156 × 96 mm^3^, 1 mm isotropic resolution, bandwidth = 240 Hz/Px).

### 2.5 Laboratory-based behavioral measure of risk taking

To confirm the effect of CR on behavior, the computerized Balloon Analogue Risk Task (Inquisit lab, Millisecond Software, LLC. United States) was administered at the end of each session to measure individual risk-taking propensity. Participants were asked to pump 30 balloons in the computer, and the average number of pumps of the non-exploded balloons was taken as the measured index.

### 2.6 MR spectral processing

All ^1^H and ^31^P MR spectra were analyzed by LCModel. ^31^P metabolite concentrations were calculated assuming [γ-ATP] of 3 mM using a basis-set composed of 13 simulated ^31^P metabolites, including PCr, Pi, α-ATP, β-ATP, γ-ATP, phosphocholine (PC), phosphoethanolamine (PE), glycerophosphocholine (GPC), glycerophosphoethanolamine (GPE), membrane phospholipids (MP), nicotinamide adenine dinucleotide hydrogen (NADH), nicotinamide adenine dinucleotide oxidized (NAD^+^), Pi (extra-cellular). Unsuppressed water spectra were used for the quantification of ^1^H metabolites with a basis set of 20 simulated spectra of metabolites including ascorbate (Asc), alanine (Ala), aspartate (Asp), creatine (Cr), phosphocreatine (PCr), glucose (Glc), glutamine (Gln), glutamate (Glu), glutathione (GSH), glycerolphosphorylcholine (GPC), phosphorylcholine (PCho), myo-inositol (Ins), lactate (Lac), N-acetylaspartate (NAA), N-acetylaspartylglutamate (NAAG), phosphorylethanolamine (PE), scyllo-inositol (Scyllo), taurine (Tau), γ-aminobutyric acid (GABA), glycine (Gly) and experimentally measured macromolecule spectra. The representative ^1^H and ^31^P MR spectra and LCModel fits were shown in Supplementary Figure S1.

### 2.7 Sample size calculation and statistical analysis

The objective of the sample size calculation was to confirm that a pre-planned sample size of 20 subjects would be sufficient to detect a similar effect size than observed in the pre-clinical and *ex-vivo* data, reporting change in brain NAD during CR (∼20% in NADH in human red blood cell (Ch et al., 2021), ∼30% in NAD^+^ in mice liver (Nakahata et al., 2009)).

From a previous study performed by our group on 25 healthy volunteers (Byrne et al., 2019), baseline brain NAD average values and standard deviation (SD) served as a basis for the power calculation. Using standard assumptions (Alpha = 0.0125, 4 tests in parallel, Bonferroni correction for multiplicity; normally distributed data, two-sided tests; AM SD equal to PM SD) we found that with 20 participants, a 6%–12% change in NAD values and a 1.4-fold change in NAD^+^/NADH ratio would be detected between AM and PM and lead to significant finding in 80% of the cases. Therefore, enrolling 25 participants was deemed appropriate to mitigate for some possible drop-out or non-interpretable data set.

To investigate whether the circadian rhythm had significant influence on the metabolite level and the behavioral performance, paired t-tests were done for each NAD^+^, NADH and their ratio, the BART results, and the cortisol level between AM and PM values. Two-tailed F-test was performed to evaluate the variance difference between AM and PM sessions (alpha = 0.05). The Spearman correlation analysis was performed for correlation test between parameters.

Exploratory analysis to detect possible changes in other brain metabolites measured by ^1^H and ^31^P MR was conducted using paired t-tests without Bonferroni correction for multiplicity.

## 3 Results

The characteristics of the 25 healthy participants are displayed in Table 1. They show a homogenous sleeping behavior as determined by the MEQ questionnaire and the wake-up time on the day of the experiment, and a stable sleep duration the week before the study. The start time of the AM session was consistently within 2 h after wake-up time.

**TABLE 1:** Characteristics of the 25 male subjects. Abbreviations: BMI: body mass index, SD: standard deviation.

Characteristics	Mean (+/- SD)
Age (year)	21.5 (2.4)
BMI (kg/m^2^)	21.9 (2.1)
Morning Evening Questionnaire	50.4 (6.4)
Mean sleep duration (h:min)	07:55 (0:34)
Wake-up time on the experimental day (h:min)	7:03 (0:22)
Time between wake-up and AM session (h)	1.6 (0.3)

**FIGURE 2:**
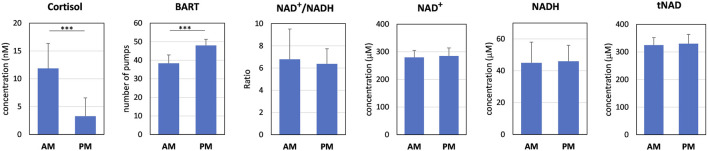
Results of the AM and PM sessions for salivary cortisol, BART test, NAD^+^/NADH redox ratio and NAD levels measured by ^31^P-MRS in the occipital region. Values are mean +/- standard deviation; ***p < 0.001.

Morning salivary cortisol level was 3.6-fold significantly greater than in the afternoon (*p* < 0.0001, Figure 2). Results of the BART test indicated a statistically significant 25% increase (*p* < 0.0001) in average number of pumps in the afternoon. Brain NAD^+^/NADH redox ratio decrease by −5.9% in the PM session but did not reach statistical significance (*p* = 0.455). NAD levels were reliably measured but without significant differences nor trends between AM and PM sessions. The initial statistical hypothesis was that we should be able to detect a 1.4 fold change for the NAD ratio and 12% change for NAD.

We noted an increase in the variability of the NAD^+^/NADH redox ratio measurement in the morning compared to the afternoon which was statistically significant (Variance_AM_ 7.4; Variance_PM_ 1.8; *p* = 0.0005). Significant differences in variance between AM and PM values were not observed for the other parameters.

Exploratory analysis to detect difference between AM and PM session were conducted for the other brain metabolites quantified by ^31^P and ^1^H-MRS (see material and method section). We found a statistically significant decrease in the PM session for Taurine brain level (Tau −5.3%, *p* = 0.016). All the other metabolites, including lactate, did not display any significant differences (see Supplementary Table S1).

Using Spearman correlation analysis between parameters in the AM or PM sessions, we could not identify any significant correlation across salivary cortisol level, BART test, and NAD values. These parameters did not correlate with wake-up time or time between wake-up time and AM session, except a weak correlation between wake-up time and BART scores in the afternoon (r = −0.4414, *p* = 0.027, see Supplementary Figure S2).

**FIGURE 3:**
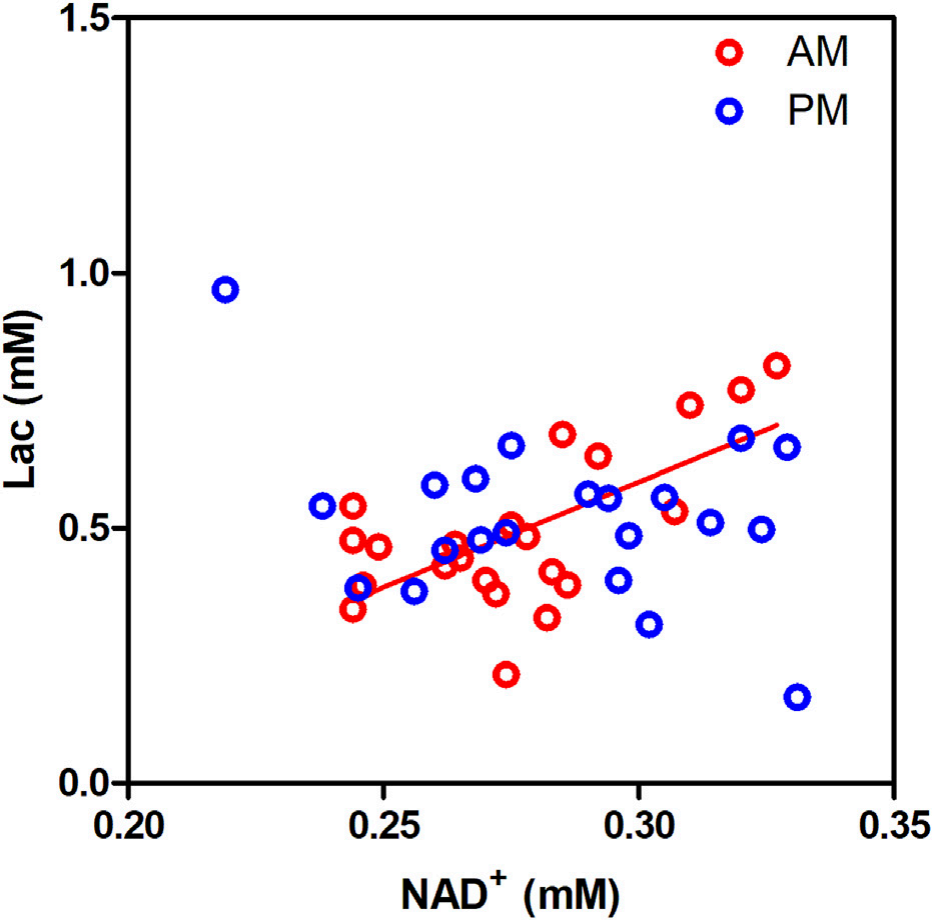
Lactate (Lac) levels correlate significantly with NAD^+^ levels in the human occipital lobe in the morning but not in the afternoon.

Because lactate dehydrogenase catalyzes the interconversion of lactate to pyruvate with the concurrent conversion of NAD^+^ to NADH, we investigated if there was an association between brain NAD and lactate. Correlation analysis between lactate and NAD^+^ levels revealed a significant correlation (Figure 3; r = 0.677, *p* = 0.0005) exclusively in the morning, while this correlation is not observed in the afternoon (r = −0.282, *p* = 0.203). No correlation was detected between lactate and NADH or the NAD^+^/NADH redox ratio.

## 4 Discussion

This is the first *in vivo* study to investigate the effect of CR on brain NAD levels and their ratio in humans. Although no significant differences in NAD^+^, NADH, and NAD^+^/NADH were detected between the morning (AM) and afternoon (PM) sessions, there was a significant variance difference in NAD^+^/NADH, with a higher variance of NAD^+^/NADH redox ratio in the morning. This study also confirms that the CR does affect risk-taking behavior, with higher performance on the task observed during the afternoon session. As expected, salivary cortisol level was also modulated by the CR, with a higher level in the morning, confirming that the participants were in two circadian different physiological states in the morning and in the afternoon. None of the other measured brain metabolites were significantly affected by the CR except for taurine.

### 4.1 Non-detectable circadian oscillation of NAD content and ratio in human occipital lobe

The levels of NAD^+^, NADH, and NAD^+^/NADH in the human occipital lobe did not show significant differences between the morning and evening. There are several plausible explanations for this observation. Firstly, the experimental time slots (8–9 a.m. and 15–16 p.m.) may not have exhibited the largest difference in NAD levels, and the small difference falls within the measurement error of NAD. Our previous test-retest reproducibility study assessed the measurement power and indicated that changes of less than 5% for NAD^+^ and 10% for NADH and NAD^+^/NADH could be detected with the current sample size (Xin et al., 2018). Therefore, the difference in NAD^+^ levels during the experimental time slots of the day might be below 5%, and for NADH and NAD ratio, the difference might be below 10%. It is worth noting that previous reports in mice liver and human red blood cells have reported approximately 30% maximum change in NAD^+^ and 20% change in NADH, respectively. Nevertheless, the extent of NAD circadian oscillation in the human cortex still remains unknown. Future studies should investigate multiple time points throughout the day to capture the maximum circadian oscillation.

Secondly, the occipital lobe may not demonstrate circadian rhythmicity in NAD levels, or NAD may not be involved in the regulation of circadian rhythms in that particular brain region. The suprachiasmatic nucleus (SCN) in the hypothalamus is considered the central circadian clock of the brain. Transcription of clock genes is associated with the redox status, and a previous study has shown the presence of redox oscillations in the SCN (Wang et al., 2012). However, it remains unknown whether redox oscillations exist in other peripheric brain regions. An fMRI study has observed circadian rhythm effects in various subcortical and cortical regions, including the primary sensorimotor cortices, occipital pole, and intraparietal sulcus (Muto et al., 2016). However, in a genome-wide 24-h rhythmicity study across seven human tissues, the brain showed the lowest rhythmicity between morning and evening gene expression (Talamanca et al., 2023), suggesting that peripheral brain region might not display a strong CR effect on metabolism.

It should be noted that the current MRS voxel used for measurement is relatively large, which may introduce partial volume effects and attenuate the circadian oscillations. Future studies should consider extending the measurements to other brain regions to investigate whether the circadian rhythm affect various metabolite levels in a brain regions specific manner.

Last, inter-subject variability in the CR might be also one potential factor contributing to the current observation. We investigated the association between NAD and mean wake-up time and did not observe any links, suggesting this might not be the main factor leading to the current result.

Another interesting finding is the circadian-dependent correlation between NAD^+^ and lactate. Although no significant differences were observed for NAD^+^ and lactate levels across the AM and PM sessions, they correlate strongly and exclusively in the morning. This could imply that over other salvage, biosynthesis and recycling pathways, lactate dehydrogenase plays a role for maintaining NAD^+^ homeostasis in the morning by recycling it from NADH (Xie et al., 2020).

### 4.2 Large variance in NAD^+^/NADH in the morning

Interestingly, we observed a circadian-dependent effect on the variance in NAD^+^/NADH ratio measurement. Specifically, we found a significantly larger variance in the NAD redox ratio in the morning compared to the afternoon. Consequently, our findings suggest that conducting redox-related metabolic studies in the afternoon may be optimal, considering the substantial variance in NAD^+^/NADH observed in the morning. The increased variance in NAD^+^/NADH levels may be attributed to the effect of fasting on ketosis. In a previous study, we demonstrated that nutritional ketosis could elevate the NAD^+^/NADH ratio in the human occipital lobe (Xin et al., 2018). Hence the known variability effect of fasting on ketosis might explain the higher variability in brain NAD^+^/NADH ratio the morning, while in the post-prandial afternoon, ketosis is at a more consistent lower level.

### 4.3 Effect of the CR on the other brain metabolite levels: only taurine

When looking at over 30 metabolites measured by ^1^H- or ^31^P-MRS (Supplementary Table S1), only taurine was found to be statistically decreased in the afternoon (−5.3%; *p* = 0.016). While this analysis was conducted as an exploratory investigation with no correction for multiplicity, it indicates that the levels of metabolites in the brain occipital region are very stable and not significantly affected by the CR. Interestingly, results from rodent studies revealed that taurine content can exhibit specific daily patterns in hypothalamic regions (Duvilanski et al., 2003), and taurine treatment modulates circadian rhythms in mice fed a high fat diet (Figueroa et al., 2016). It was also shown that taurine deficiency, including non-human primate brain levels, correlated with poor health in aging (Singh et al., 2023). Further CR studies should consider the prospective measurement of taurine levels in different regions of the human brain and explore how taurine supplements could impact brain CR metabolism in health and diseases.

### 4.4 Limitation of the study

Circadian behavioral and physiological rhythm can be susceptible to various factors, such as age, gender, sleep, physical exercise, alcohol use, and smoking. To mitigate the potential impact of age, gender, and sleep on circadian rhythms, our study focused exclusively on a homogenous group of male participants aged 18–40 years old, who exhibited consistent sleep behaviors. However, it is worth noting that we did not collect data on factors like smoking, alcohol consumption, and other substance use (Meyrel et al., 2020; Wang et al., 2023), which have been shown to disrupt circadian rhythm. In this study, we assessed cortisol levels in both the morning and afternoon and found that a typical circadian rhythm was observed. This suggests that these factors may not exert a significant influence on our study cohort. However, future studies should consider incorporating such information to mitigate the potential effects on metabolism associated with circadian rhythm. The scope of this study should be extended in the future by including a larger and more diverse cohort, encompassing both males and females and spanning a wider range of age groups. This will enable us to gather more comprehensive data on circadian NAD dynamics for a broader population. Furthermore, due to the intrinsically low sensitivity of ^31^P-MRS, measuring NAD levels in the suprachiasmatic nucleus *in vivo* remains a considerable challenge. Consequently, we were unable to assess NAD dynamics in this central brain region of regulating circadian rhythms.

## 5 Conclusion

In conclusion, we have shown that there is no statistically significant effect of the CR on occipital brain NAD levels in humans. The other brain metabolites measured, including lactate, were not significantly affected by the CR either, except for taurine. The CR did impact risk-taking behavior and salivary cortisol level, confirming that the participants were in two circadian different behavioral and physiological states in the morning and in the afternoon. Measurement of the CR effect on NAD, lactate and taurine in other brain regions might provide stronger effects.

## Data Availability

The original contributions presented in the study are included in the article/Supplementary Material, further inquiries can be directed to the corresponding author.
