# Supplementary material for: Effect of circadian rhythm on NAD and other metabolites in human brain
Source: Front Physiol. 2023 Nov 9;14:1285776. doi: 10.3389/fphys.2023.1285776 (PMC10665902; doi:10.3389/fphys.2023.1285776)
Supplement: Supplementary file 1 [file DataSheet1.PDF]

Supplementary Material

Effect of Circadian Rhythm on NAD and Other Metabolites in Human Brain

Bernard Cuenoud*, Zhiwei Huang, Mickael Hartweg, Mark Widmaier, SongI Lim, Daniel Wenz, Lijing Xin*

*** Correspondence:** Bernard Cuenoud: Bernard.cuenoud@nestle.com

Figure S1. Representative ^{1}H (right) and ^{31}P (left) MR spectra of the human occipital lobe, and the corresponding LCModel fits.

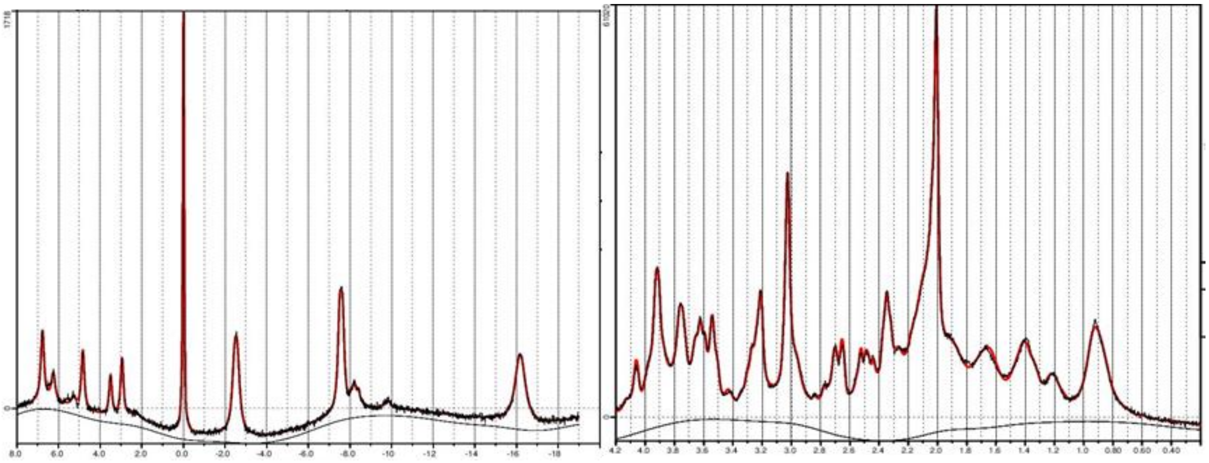

Figure S2. Correlation between wake-up time of the experimental day and the mean BART trials in the afternoon (the line indicates the linear regression).

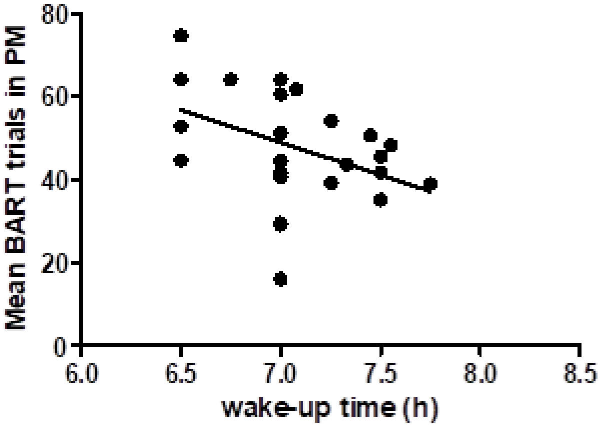

Table S1. Mean metabolite levels (mM) +/- standard deviation (SD), physiological parameters, spectral linewidth (LW), and SNR of ^{31}P and ^{1}H MRS data in the morning (AM) and in the afternoon (PM). *p* is T-test between AM and PM values.

	AM		PM		
**^{31}P MRS**	**Mean**	**SD**	**Mean**	**SD**	***p***
**PCr**	3.18	0.21	3.15	0.28	0.646
**Pi**	0.89	0.06	0.89	0.06	0.796
**PC**	0.60	0.08	0.63	0.09	0.167
**PE**	1.40	0.12	1.41	0.12	0.860
**GPC**	1.00	0.10	1.00	0.09	0.905
**GPE**	0.63	0.07	0.63	0.05	0.844
**MP**	0.40	0.15	0.45	0.17	0.086
**NADH**	0.05	0.01	0.05	0.01	0.790
**NAD^+**	0.28	0.03	0.28	0.03	0.577
**Pi(ext)**	0.14	0.07	0.13	0.06	0.885

**UDPG**	0.18	0.02	0.19	0.02	0.153
**tNAD**	0.33	0.03	0.33	0.03	0.547
**NAD^+/NADH**	6.78	2.73	6.38	1.35	0.455
**pH (intracellular)**	6.99	0.01	6.99	0.01	0.947
**pH (extracellular)**	7.39	0.01	7.40	0.01	0.105
**Mg^{2+}**	0.17	0.00	0.17	0.00	0.102
**k_{CK} (s^{-1})**	0.34	0.04	0.37	0.13	0.330
**^{1}H MRS**	**Mean**	**SD**	**Mean**	**SD**	***p***
**Ala**	0.40	0.34	0.43	0.50	0.582
**Asp**	1.80	0.36	1.89	0.44	0.304
**GABA**	0.86	0.24	0.80	0.23	0.203
**Gln**	1.72	0.31	1.75	0.39	0.542
**Glu**	6.85	0.62	7.04	0.67	0.124
**GSH**	0.96	0.19	0.96	0.20	0.979
**Gly**	0.24	0.12	0.21	0.11	0.169
**Ins**	5.15	0.78	5.18	0.66	0.683
**Lac**	0.49	0.15	0.59	0.36	0.183
**NAA**	9.24	0.76	9.20	0.64	0.687
**Scyllo**	0.14	0.09	0.15	0.10	0.418
**Tau**	1.16	0.17	1.10	0.21	**0.016**
**NAAG**	1.34	0.24	1.34	0.23	0.975

**PE**	1.66	0.16	1.73	0.19	0.135
**NAA+NAAG**	10.59	0.83	10.54	0.74	0.700
**Glu+Gln**	8.57	0.75	8.80	0.82	0.185
**GPC+PCho**	0.73	0.12	0.73	0.11	0.858
**Cr+PCr**	6.39	0.39	6.32	0.39	0.378
**Gly+Ins**	5.39	0.72	5.37	0.62	0.760
**Spectral quality**	**Mean**	**SD**	**Mean**	**SD**	***p***
**LW (^{31}P, Hz)**	14.7	1.4	14.4	1.6	0.288
**SNR (^{31}P, -)**	87.2	21.4	90.5	19.0	0.649
**LW (^{1}H, Hz)**	13.5	0.8	13.1	0.4	**0.042**
**SNR (^{1}H, -)**	73.7	11.0	74.7	8.0	0.649
